# Echocardiographic characteristics of transcatheter closure of patent foramen ovale with mallow biodegradable occluder: A single-center, phase III clinical study

**DOI:** 10.3389/fcvm.2022.945275

**Published:** 2022-08-12

**Authors:** Lin Song, Peixuan Shi, Xiaozhou Zheng, Li Hongxin, Ziang Li, Meng Lv, Haiyan Wang

**Affiliations:** ^1^Department of Medical Ultrasound, Shandong Medicine and Health Key Laboratory of Abdominal Medical Imaging, The First Affiliated Hospital of Shandong First Medical University & Shandong Provincial Qianfoshan Hospital, Jinan, China; ^2^Department of Medical Ultrasound, Shandong Provincial Qianfoshan Hospital, Shandong First Medical University, Jinan, China; ^3^Department of Cardiovascular Surgery, Shandong Engineering Research Center for Health Transplant and Material, The First Affiliated Hospital of Shandong First Medical University & Shandong Provincial Qianfoshan Hospital, Jinan, China; ^4^Department of Anesthesiology, The First Affiliated Hospital of Shandong First Medical University & Shandong Provincial Qianfoshan Hospital, Jinan, China

**Keywords:** patent foramen ovale, biodegradable occluder, transthoracic echocardiography, right-to-left shunt, transcatheter closure

## Abstract

**Background:**

Transcatheter occlusion of patent foramen ovale (PFO) has become a recognized treatment option for high-risk PFO-related diseases. However, traditional metal occluders have some disadvantages, such as permanent retention in the body, abrasion of tissues, and obstruction of access to the left side of the heart for interventional procedures. With biodegradable occluders that release non-toxic degradation products and are absorbable by the body, the risk of long-term complications could be greatly reduced. The experimental results of using a PFO-degradable occluder in beagle dogs in early stages, independently developed by Shanghai Mallow Medical Instrument Co., Ltd., showed that the occluding umbrella disc network was degraded 6 months after occlusion. The occluder also showed good memory, biocompatibility, and mechanical properties.

**Methods:**

As one of the multi-center research units, this prospective Phase III clinical trial study included 16 patients with PFO-related complications who were treated with a degradable occluder. The follow-up period lasted for 12 months to analyze the echocardiographic characteristics and procedural feasibility.

**Results:**

The immediate success rate of the procedure was 100% with no serious complications. Postoperative color Doppler transthoracic echocardiography (TTE) and transesophageal echocardiography (TEE) at 12 months showed that one patient with atrial septal aneurysm (ASA) had a residual shunt at the edge of the occluder, and contrast transcranial Doppler (cTCD) showed that all patients were grade I or 0 right-to-left shunts (RLS), indicating that the occlusion success rate was 100%. The occluder gradually degraded after the procedure, particularly when the umbrella disc structure became vague, and the size of the occluder decreased significantly 6 months after occlusion.

**Conclusions:**

PFO closure with a Mallow degradable occluder has a high plugging success rate, is safe and effective, and has no serious complications. However, for PFO closure with special anatomical features, further research with a larger sample size is required. TTE can dynamically, conveniently, and accurately observe the entire degradation process of the occluder.

**Clinical Trial Registration:**

ChiCTR1900024036.

## Background

During the embryonic period, the foramen ovale is a part of the normal fetal circulatory system and is an important physiological channel for fetal survival. After birth, the right atrial pressure decreases to less than the left atrial pressure, resulting in functional closure of the foramen ovale. If it remains patent beyond 3 years of age, it is called patent foramen ovale (PFO). In recent years, an increasing number of studies have found that PFO may cause occult ischemic stroke, migraine, recurrent transient ischemic attack (TIA), decompression sickness, syncope, temporary aphasia, obstructive hypopnea-apnea syndrome, supine dyspnea, platypnea-orthodeoxia syndrome, pulmonary hypertension, refractory hypoxemia in cases of right ventricular myocardial infarction, etc. (1–3). Whether PFO requires treatment is still controversial. However, for PFOs with high-risk conditions, active treatment is generally recommended, and transcatheter closure has become the preferred option.

In recent years, research regarding PFO and unexplained ischemic stroke worldwide has progressed rapidly, and several countries have issued or updated guidelines for the secondary prevention of stroke. The recommendation for transcatheter occlusion of the foramen ovale has progressively increased to level I, with a level of evidence A, in patients with PFO combined with a medium-large right-to-left shunt (4–9). PFO closure in patients with migraine has also been actively studied (10, 11), with many reports demonstrating benefits from occlusion (12, 13).

Currently, metallic PFO occluders are widely used in clinical practice. Once the metallic occluder is implanted in the body, it may cause wearing of adjacent structures, thrombosis, hemolysis, nickel allergy, cardiac conduction abnormalities, and obstruction of the transseptal access to the left atrium for future treatment of left-sided heart diseases (transcatheter mitral valve intervention, left atrial appendage occlusion, pulmonary vein angioplasty, and atrial arrhythmia ablation) in the long-term. An optimal occluder serves as a temporary bridge for cardiac self-repair, and the task is accomplished once the defect is fully endothelialized and covered by newly formed autologous tissue. Therefore, if the occluder is composed of non-toxic, biodegradable materials, the risk of long-term complications can be greatly reduced.

The PFO-degradable occluder, independently developed by Shanghai Mallow Medical Instrument Co., Ltd., is composed of an occluding umbrella disc, interlayer membrane, and suture, which is composed of degradable woven silk. The interlayer membrane is composed of a polyethylene terephthalate (PET) matrix membrane and the suture is made of degradable nylon thread. The results of previous beagle dog animal experiments showed that the umbrella disc of the occluder was degraded 6 months after occlusion, and the occluder possessed good memory, biocompatibility, and mechanical properties. This study aimed to observe the perioperative and near-mid-term postoperative echocardiographic characteristics, procedural success, and patient safety in one of the medical centers participating in the Phase III clinical trial of the Mallow biodegradable PFO occluder in China.

## Materials and methods

### Study population

We selected a total of sixteen patients who had been diagnosed with PFO and admitted to the Department of Cardiovascular Surgery of the First Affiliated Hospital of Shandong First Medical University (Shandong Provincial Qianfoshan Hospital) between June and August 2020. The enrollment criteria were as follows: (1) PFO diagnosed with a medium-large right-to-left shunt (RLS) by imaging; (2) age ≥ 16 years; (3) voluntary participation in human clinical trials with signed informed consent; and (4) occult ischemic stroke, migraine, or unexplained dizziness. The patients were comprehensively evaluated by a multidisciplinary team, including cardiovascular surgery, interventional department, neurology, and echocardiography.

The exclusion criteria were as follows: (1) cerebral embolism in which an etiology could be found; (2) migraine of other known causes, psychosomatic disorders, or cognitive impairment that prevented the completion of the questionnaire; (3) contraindication to antiplatelet or anticoagulation therapy; (4) inferior vena cava or pelvic vein thrombosis, infection, sepsis, or intracardiac thrombosis; (5) pregnancy or possible pregnancy during the trial; (6) combined pulmonary hypertension or PFO as a survival channel; and (7) massive cerebral infarction within 4 weeks.

This study was approved by the Ethics Committee of the First Affiliated Hospital of Shandong First Medical University (2020 [QX-C001]), and all study participants provided informed consent.

### Methods

#### Patients with cerebral infarction received a risk of paradoxical embolism score

Firstly, PFO-related stroke was exclusively diagnosed. Then, the RoPE scores were used to determine the probability of PFO-related stroke and for the preoperative assessment of PFO occlusion. The RoPE score was calculated as follows: no history of hypertension (1 point); no history of diabetes (1 point); no history of stroke or TIA (1 point); non-smoking (1 point); cortical infarct on imaging (1 point); an age of 18–29 (5 points), 30–39 (4 points), 40–49 (3 points), 50–59 (2 points), 60–69 (1 point), and 70 years or older (0 points). With RoPE scores of 7, 8, and 9, the PFO attributable scores for stroke were 72, 84, and 88%, respectively, indicating possible benefit from PFO closure.

#### Headache impact test (HIT-6 Score) for patients with migraine

The HIT-6 score was used to evaluate the impact of headaches on the patients' work, family, and social activities. The scale consists of six questions that evaluate the impact of headaches within the previous 4 weeks. The HIT-6 score (36–78 points) divides the effect of headache into four grades: no or mild (36–49 points), moderate (50–55 points), significant (56–59 points), and severe effect (60–78 points).

#### PFO biodegradable occluder device

**Figure 1:**
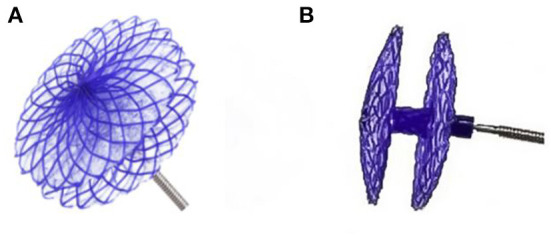
Biodegradable occluder. **(A)** The positive view of the occluder; **(B)** side view of the occluder.

The occluder consists of a blocking umbrella disc, interlay membrane, and suture. It is a double-disc, single-rivet shape, with umbrella discs, waist, and right disc rivets made of polydioxanone (PDO). The degradation products are carbon dioxide and water, the internal interlay membrane is a PET polyester film, and the suture is a biodegradable nylon thread. The occluder is currently available in seven sizes: 18/18, 24/18, 24/24, 30/24, 30/30, 34/24, and 34/34 mm for the right and left atrial discs. Occluders of different sizes have different waist heights (3, 4.5, and 5.5 mm) (Figure 1).

#### Research process

The trial course was ≥ 12 months with a preoperative diagnostic flow involving transthoracic echocardiography (TTE)–contrast TTE (cTTE)/contrast transcranial Doppler (cTCD)–transesophageal echocardiography (TEE)–contrast TEE (cTEE), intraoperative occluder delivery and release under X-ray and TTE monitoring or TEE guidance alone. An immediate efficacy evaluation was conducted after occlusion. TTE was performed at 48 h, 1 month, and 3 months postoperatively, TTE and cTCD were performed at 6 months postoperatively, and TTE and cTCD follow-up occurred at 12 months postoperatively. For precise diagnosis and treatment, we added TEE examination at 12 months postoperatively to the original study design to observe the residual morphology of the occluder and RLS color shunt signal after obtaining the consent of Mallow Medical Co., Ltd., and the patients. Additionally, routine blood and urine tests, blood biochemistry, coagulation function tests, and electrocardiograms were routinely performed and observed at the aforementioned time points.

### Preoperative diagnostic criteria for PFO imaging

#### Echocardiogram

Patent foramen ovale was mainly diagnosed using TTE, cTTE, TEE, cTEE, and cTCD before occlusion. Perioperative and postoperative routine follow-ups were performed using a Philips EPIQ 7C color ultrasound diagnostic instrument (Origin, USA). For TTE examination, two-dimensional (2D) echocardiography was used to scan the anatomical structure of the atrial septum, and color Doppler was used to observe the horizontal oblique left-to-right shunt in the central part of the atrial septum on the parasternal aortic short-axis view, the apical 4-chamber view or the apical 5-chamber view, and the subcostal biatrial view. RLS was mainly assessed using cTTE or cTEE. Contrast preparation and injection were as follows: blood-added activated normal saline (1 ml of autologous blood + 1 ml of air + 8 ml of normal saline) was used as a contrast agent and injected into the left median elbow vein in a pellet-type regiment according to the recommendations of the 2017 “Chinese Expert Consensus on Prophylactic Occlusion of PFO” (14). The patients assumed a left lateral decubitus position, and the number of microbubbles that developed in the left cardiac chamber within 3–5 cardiac cycles after the right heart progressed to a resting state and after the modified Valsalva maneuver (during which a manometer was used to blow raising the chest pressure to ≥ 40 mmHg [1 mmHg = 0.1333 kPa] for 10 s) under the apical 4-chamber view was used to determine the amount of RLS. RLS classification was determined by the number of microbubbles present in the left cardiac chamber on a single frame image: grade 0, no microbubbles or RLS; grade I, <10 microbubbles, a small amount of RLS; grade II, 10–30 microbubbles, a medium amount of RLS; and grade III, > 30 microbubbles or the left cardiac chamber was almost filled with microbubbles, giving a cloudy appearance, a large amount of RLS. TEE was performed in the awake and fasting state under esophageal and pharyngeal mucosal anesthesia, and the anatomical structure of the PFO and atrial horizontal shunt were observed by multiplanar 2D and color Doppler ultrasonography, and the size of the left and right atrial sides of the PFO, as well as the overlapping length of the secondary septum and primary septum (i.e., the length of the tunnel), was measured. Whether PFO is accompanied with high-risk situations such as many small defects in the left atrial side, ASA, large atrial septal motion, inferior vena cava valve or Chiari's network, secondary septal hypertrophy, and aortic root dilatation. cTEE was performed using the superior/inferior vena cava view to observe the RLS at rest and after performing a cough or breath-hold maneuver. Although some studies suggested that the grading criteria for cTEE to determine RLS were the same as those for cTTE, in our study, cTEE alone was used as a means of detecting the source of RLS due to the difficulty of performing TEE with the standard modified Valsalva maneuver in awake patients.

#### Contrast transcranial doppler

An 8080 TCD cerebral blood flow examination instrument (EME, Ltd., USA) was used. The probe was placed in the temporal window using the dual-channel single-depth technique to visualize the middle cerebral artery on that side. The configuration of activated normal saline was the same as that used in cTTE. The presence of a micro-embolic signal within 10–20 s was used as a positive criterion both in the resting state and during the Valsalva maneuver and was graded according to the number of micro-embolic signals observed: grade 0, no signals; grade I, 1–10 signals, a small amount of shunt; grade II, 11–25 signals, a moderate amount of shunt; grade III, >25 signals, a large amount of shunt, and the signals could be in the shape of a rain curtain or shower (15). The RLS included intrinsic shunts that were present in the resting state and potential shunts that appeared only when provoked by the Valsalva maneuver.

#### The procedure

Aspirin (3–5 mg/kg/d) and clopidogrel (75 mg/d) were administered orally 48 h before occlusion. Under local anesthesia for X-ray and TTE monitoring or general anesthesia for TEE monitoring alone, the delivery and release of the occluder were performed. For the blocking process, (1) to establish access for blocker implantation, the femoral vein was routinely punctured and an arterial sheath was delivered. Heparin (100 u/kg) was intravenously injected and a right cardiac catheter was passed through the PFO into the left atrium and left superior pulmonary vein. An exchange guidewire was placed in the left pulmonary vein. Depending on the size of the occluder, different delivery sheaths were selected and placed in the left atrium or at the opening of the left pulmonary vein under the guidance of the guidewire. (2) For implantation, the occluding device was immersed in the contrast agent for 5–10 min, the delivery steel cable was connected to the occluding device through the loader, the steel cable was fixed, and the occluding device was rotated clockwise to stably connect itself with the delivery steel cable. We introduced the occluding device and loader into normal saline, repeatedly exhausted, and completely pulled the occluding device into the loader, connected the loader with a sheath, and pushed a delivery steel cable to advance the occluding device to the left atrium through the sheath. (3) Under X-ray and TTE monitoring or simple TEE guidance, the release of the left umbrella disc of the occluder into the left atrium was done on parasternal aortic short-axis view, apical 4-chamber view, and subcostal biatrial view. The delivery sheath was withdrawn to retract the left umbrella disc to the atrial septum, the right umbrella disc was released on the right atrial side, and the delivery cable was repeatedly shaken to ensure safe positioning of the occluder. After the descending traction test was conducted under ultrasound monitoring, the delivery cable handle was rotated in a counterclockwise direction after the shape and position of the occluder were clearly ascertained, the occluder was released, and the delivery cable and sheath were withdrawn. Echocardiography was performed immediately after occlusion to evaluate its efficacy. The morphology and location of the occluder were observed, together with the presence of thrombosis on the surface of the occluder, the effect of the occluder on surrounding structures, pericardial effusion, and residual shunt on color Doppler ultrasonography. After occlusion, patients received routine heparin anticoagulation for 48 h, oral aspirin 3-5 mg/(kg·d) for 6 months, and clopidogrel 75 mg/d for 3 months.

### Postoperative echocardiography and contrast TCD evaluation

The morphology and location of the occluder, size of the umbrella disc, size of the ASA, and presence of a residual shunt were evaluated by TTE at 48 h, 1 month, 3, 6, and 12 months after the procedure. Residual RLS was detected using cTCD 6 and 12 months postoperatively. Twelve months after the operation, TEE was performed to observe morphological changes in the occluder and the presence of a residual shunt.

### Clinical endpoints and criteria for successful PFO closure

Clinical follow-up at 12 months after occlusion was used as the clinical study endpoint, and the success rate of device release in the immediate postoperative period, the success rate of blocking at 48 h, 1 month, 3, 6, and 12 months after occlusion, and the overall success rate at 12 months after occlusion were used as the evaluation indices for effectiveness. The incidence of complications during the follow-up period at each of the aforementioned time points was used as the safety evaluation index.

The success of closure at 48 h, 1 month, and 3 months postoperatively was defined as normal occluder morphology and position on TTE, no thrombus on the occluder, no effect on adjacent tissues, and no left-to-right shunt at the atrial level. The same TTE observation was performed at 6 and 12 months postoperatively, with normal occluder morphology and position, and no or mild RLS by cTCD.

### Statistical analysis

Data analysis was performed using SPSS 20.0. Continuous variables with a normal distribution were expressed as mean ± standard deviation. Pairwise comparisons were performed with the Student's *t*-test, and repeated measures analysis of variance was used to compare the relevant sample data of multiple groups with normal distribution. When the number of cases with measurement data in a subgroup was small, a non-parametric test was performed. For variables with a non-normal distribution, median, and 25th and 75th percentiles (interquartile range) were used for representation, and pairwise comparisons were made using the Mann–Whitney *U*-test. The Chi-squared test (R × C contingency table) was used to compare constituent ratios between the groups. Statistical significance was set at *p* < 0.05.

## Results

### Patient baseline characteristics

Altogether 16 patients (9 women and 7 men) were admitted to our hospital from June to August 2020, with a mean age of 45.69 ± 16.21 years. Among these, 5 patients had an occult ischemic stroke. Case 13 had middle cerebral artery stenosis; however, the infarction was located in both cerebellar hemispheres and the right occipital lobe, which was not related to middle cerebral artery stenosis seen on imaging. Since no other reason accounted for cerebral infarction in this patient, we suspected that the preoperative infarction might have resulted from a paradoxical embolism caused by a PFO. Therefore, we included the patient in this study. Twelve patients had migraine (cases 4, 6, and 16 with combined occult stroke; case 9 with a missing field of view; and cases 2, 8, 9, 11, and 12 with aura migraine) and 2 patients experienced dizziness. Eighty percent (4/5) of the patients with cerebral infarction had a RoPE score ≥ 7. The mean preoperative HIT-6 score in patients with migraine was 58.3 ± 14.4. Other medical conditions included hypertension (*n* = 2), diabetes mellitus (*n* = 2), hyperlipidemia (*n* = 1), smoking history (*n* = 1), and drinking history (*n* = 4). None of the patients had coronary heart disease (CHD), chronic kidney disease (CKD), or chronic obstructive pulmonary disease (COPD) (Table 1).

**Table 1:** Baseline demographic characteristics of the study population.

**NO**	**Sex**	**Age (years)**	**migraine**	**stroke**	**Color doppler by TTE**	**cTCD**	**cTTE**	**PFO size of left atrial side by TEE(mm)**	**PFO size of right atrial side by TEE(mm)**	**PFO length of tunnel by TEE(mm)**	**Color doppler by TEE**	**Secondary septal thickness (mm)**	**cTEE**	**RLS by cTEE**	**ASA**	**Occluder size (mm)**	**Personal history, combined diseases**
1	W	59	NO	NO	Yes	-	R – V III	1.3	1.1	4	Yes	3	R – V +	Cluster	NO	24/24	-
2	W	33	Yes	NO	Yes	-	R III V III	1.5	6.3	20	Yes	3.0	R + V +	Cluster	NO	24/24	-
3	W	28	Yes	NO	NO	-	R – V III	1.0	3.1	12	Yes	3.6	R + V +	sparse	Yes	30/30	-
4	M	55	Yes	Yes	NO	-	R III V III	2.0	4.0	14	Yes	5.0	R + V +	Cluster	Yes	30/30	Drink
5	M	33	NO	Yes	NO	R III V III	-	1.5	3.0	10	Yes	5.5	R – V +	sparse	NO	24/24	-
6	W	67	Yes	Yes	NO	R III V III	R II V III	3.7	13	11	Yes	7.9	R – V +	Cluster	Yes	24/24	HTN, DM
7	W	42	Yes	NO	NO	R III V III	-	2.7	4.2	6	Yes	4.0	R – V +	sparse	Yes	34/34	-
8	M	58	Yes	NO	NO	-	R III V III	1.5	4.0	6	Yes	4.8	R – V +	Cluster	NO	24/24	-
9	W	21	Yes, Visual field loss	NO	NO	-	R III V III	3.0	5.0	10	Yes	3.0	R + V +	Cluster	NO	24/24	-
10	W	29	Yes	NO	NO	-	R III V III	1.9	5.7	6.8	Yes	3.0	R + V +	Cluster	NO	24/24	-
11	M	59	Yes	NO	NO	-	R III V III	4.0	5.3	11.5	Yes	6.0	R – V +	Cluster	Yes	24/24	Drink, smoke
12	M	28	Yes	NO	NO	-	R – V II	0.6	1.5	12	NO	4.0	R + V +	sparse	NO	24/24	-
13	M	55	NO	Yes	NO	R III V III	-	2.5	8.0	7.6	Yes	4.4	R + V +	Cluster	NO	24/24	DM, HPL,drink
14	W	33	Yes	NO	Yes	-	R III V III	2.5	3.3	9.6	Yes	2.4	R + V +	Cluster	NO	24/24	-
15	W	63	NO	NO	Yes	-	R – V III	2.0	5.0	8.3	Yes	5.7	R + V +	Cluster	NO	24/24	HTN
16	M	68	Yes	Yes	NO	-	R – V III	1.0	2.3	12	Yes	4.5	R – V +	sparse	NO	24/24	Drink

*R stands for resting-state; V stands for after Valsalva maneuver; DM, Diabetes Mellitus; HTN, Hypertension; HPL, Hyperlipidemia*.

### Preoperative PFO imaging features

#### Transthoracic echocardiography

(1) Color Doppler revealed left-to-right shunt in four cases of PFO (cases 1, 2, 14, 15); (2) PFO combined with ASA was seen in five cases (cases 3, 4, 6, 7, and 11) (Table 1).

#### Contrast TTE /contrast TCD

In case 6, both cTCD and cTTE were performed preoperatively. cTCD showed intrinsic RLS (classified as grade III), while cTTE demonstrated grade II and grade III RLS at rest and after Valsalva maneuver, respectively. Cases 5,7, and 13 had undergone cTCD examination alone, all of which were found to have grade III intrinsic RLS. The remaining 12 patients underwent cTTE alone. In the resting state, RLS was grade 0 in 5 cases, grade II in 0 cases, and grade III in 7 cases. After the Valsalva maneuver, there was one case with Grade II and 11 cases with Grade III RLS (Table 1).

#### TEE and contrast TEE

2D TEE revealed 11 cases with overlapping tunnel lengths of ≥8 mm in the primary and secondary septa, 14 cases with primary and secondary septum spacing ≥2 mm during the Valsalva maneuver, 5 cases with ASA (ASA diagnostic criteria included atrial septal bulge amplitude > 10 mm, basal width > 15 mm, or Left and right swing amplitude of atrial septal ≥ 11–15 mm), 1 case with aortic root internal diameter ≥37 mm, and no cases with secondary septum thickness ≥10 mm or obvious eustachian valve or Chiari's network. Color Doppler TEE revealed 15 cases with a left-to-right shunt through the foramen ovale. cTEE revealed 9 cases of microbubbles entering the left atrium through the foramen ovale at rest and 16 cases of microbubbles entering the left atrium through the foramen ovale after performing the Valsalva maneuver, of which 11 cases showed a large number of clusters (Table 1).

#### Interventional closure procedures and complications associated with in-hospital procedures

Cases 12 and 14 underwent percutaneous closure of the PFO *via* a non-X-ray TEE-guided pathway under general anesthesia, while the remaining 14 patients underwent percutaneous closure *via* X-ray and TTE-guided pathways under local anesthesia. The size of the PFO occluder was selected according to TEE results. Due to large ASA, 30/30, 30/30, and 34/34 mm occluders were implanted in three ASA patients (cases 3, 4, and 7), attempting to cover the whole ASA as much as possible with the umbrella disc to avoid the risk of occluder shifting or falling off with the swing of ASA. Cases 6 and 11 just met the ASA diagnostic criteria; therefore, 24/24 mm occluders were implanted. In the remaining 11 patients, a 24/24 mm occluder was implanted (Table 1). The immediate success rate of occlusion was 100% and none showed any intracardiac thrombosis, arrhythmia, or other complications. There were no in-hospital deaths, recurrent diseases, vascular complications, or new arrhythmias after occlusion.

### Postoperative serious adverse events

None of the 16 patients were lost to follow-up, and all had regular outpatient follow-ups according to the trial procedures. One year after the operation, there was no wearing of adjacent structures, thrombosis of the occluder, shedding of the occluder, new arrhythmias, hematoma of the vascular access, etc.

Magnetic resonance imaging (MRI) scans were performed to determine the etiology of adverse effects based on the presence of embolic signs and symptoms. Serious adverse events (SAE) occurred in two cases after occlusion. However, both were considered to be unrelated to PFO occlusion. Specifically, case 10 had no obvious cause of sudden-onset slurred speech with dizziness 3 months after occlusion. No obvious infarct focus was noted on cranial MRI, and the symptoms disappeared 3 h after the onset with no sequelae. About clinical consideration, case 10 might have been related to its vasculitis. Case 13, who previously had cerebral infarction, was admitted 8 months after occlusion for “dysphonia and cough on drinking liquids for the last 1 month.” Cranial MRI revealed severe stenosis of the right middle cerebral artery, and a diagnosis of recurrent cerebral infarction was made.

### Postoperative medication

All patients received oral aspirin 3–5 mg/(kg · d) for 6 months and clopidogrel 75 mg/d for 3 months postoperatively as planned without any bleeding complications.

#### Laboratory results after plugging

Compared to the pre-occlusion state, there were no obvious abnormalities in routine blood and urine tests, blood biochemistry, and coagulation function. Electrocardiograms (ECG) showed no new arrhythmia.

### Improvement of clinical symptoms after the operation

**Figure 2:**
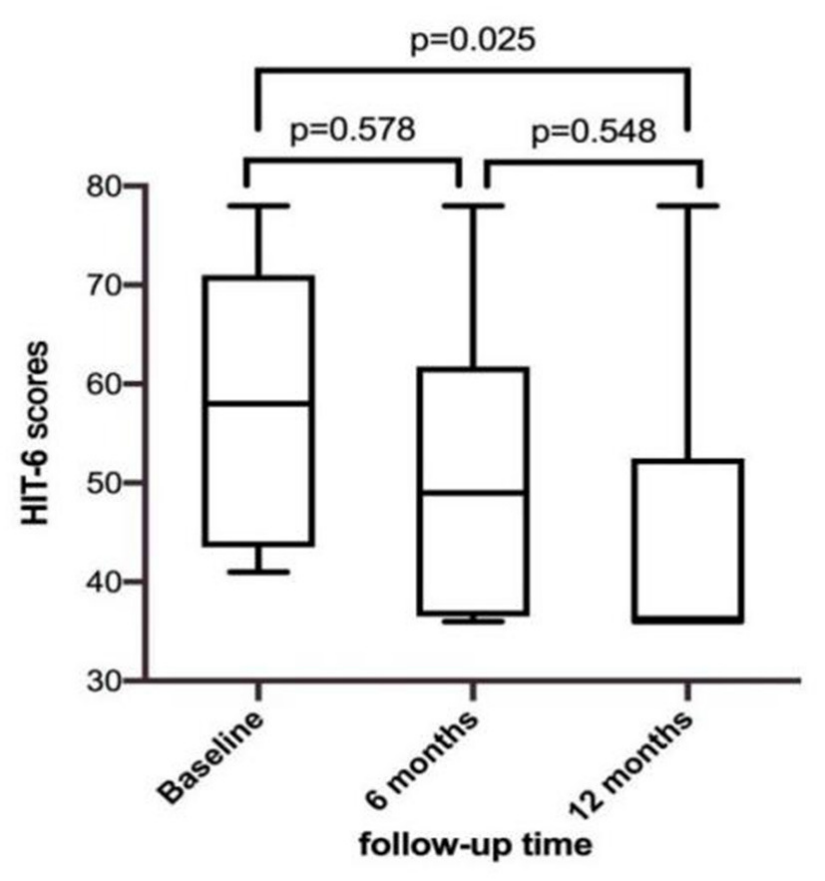
Comparison of HIT-6 score in patients with migraine before and after occlusion.

HIT-6 scores of 12 migraine patients were 50.9 ± 15.3, 48.3 ± 14.0, 51.9 ± 15.6, and 44.8 ± 14.2 at 1, 3, 6, and 12 months after the operation, respectively. There were no significant differences between the scores at 6 months postoperatively and preoperatively, and between those at 12 and 6 months postoperatively. However, there was a significant difference between the HIT-6 scores at 12 months postoperatively and preoperatively (*p* < 0.05) (Figure 2). Patients 4, 6, 9, and 11 had complete remission of migraine at 12 months after occlusion, with a complete remission rate of 33.3% (4/12). The frequency and degree of migraine attacks improved in six patients, with overall treatment efficacy of 83.3% (10/12). Except for cases 10 and 12, migraine improved after occlusion in all regardless of aura symptoms.

In case 13, cerebral infarction recurred 8 months after occlusion in the parietal lobe. This could have been related to severe stenosis of the middle cerebral artery rather than the occluding device. Furthermore, after the procedure, symptoms were significantly controlled in two patients with dizziness.

### Postoperative imaging characteristics

#### Anatomical features

##### Transthoracic echocardiography

The overall morphology of the occluder remained intact at 48 h, 1 month, and 3 months after occlusion.

**Figure 3:**
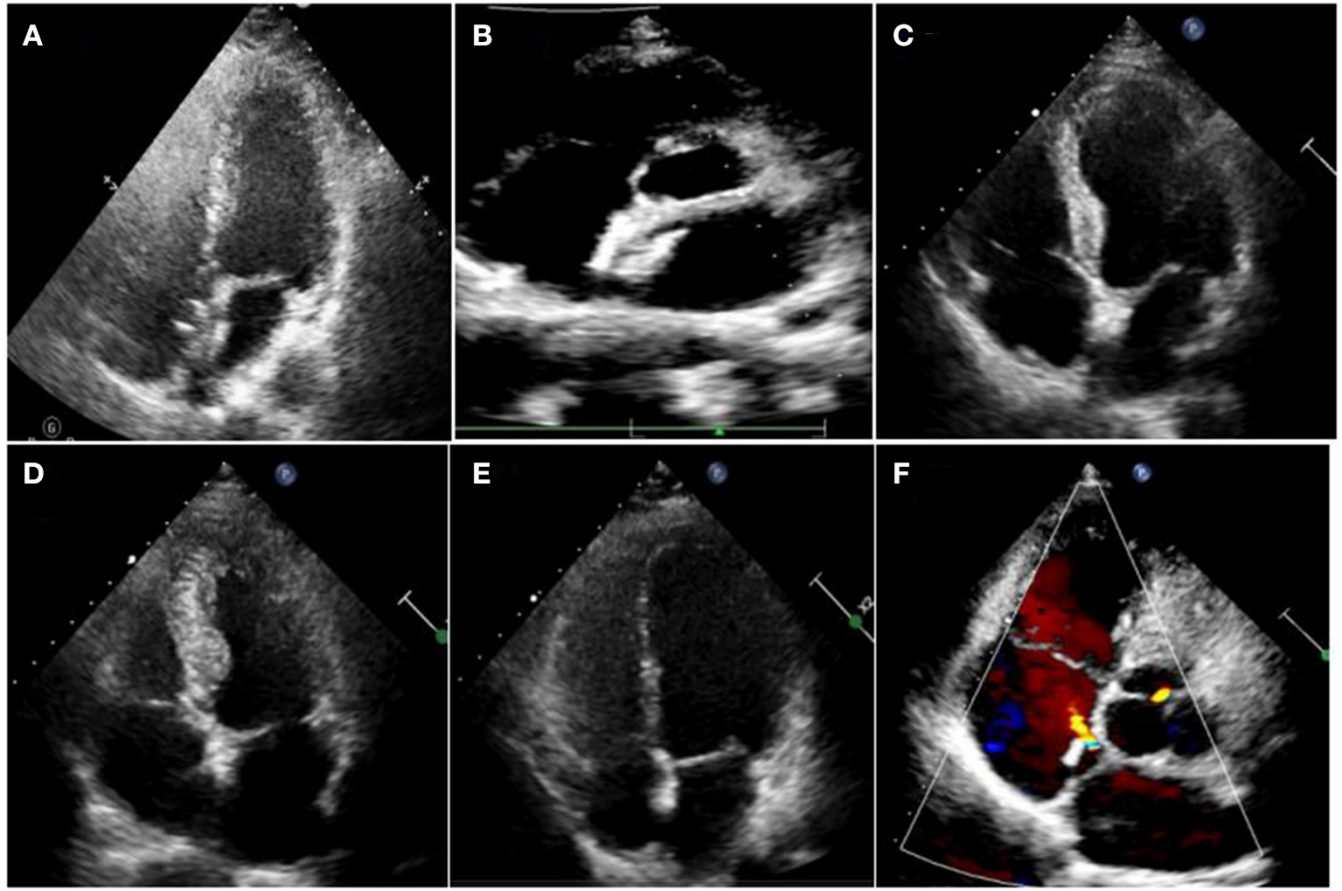
Degradation process of the occluder at different time points after occlusion. **(A)** 2D-TTE displaying the morphology of the occluder at 2 days; **(B)** 2D-TTE displaying the morphology of the occluder at 1 month; **(C)** 2D-TTE displaying the morphology of the occluder at 3 months; **(D)** 2D-TTE displaying the morphology of the occluder at 6 months; **(E)** 2D-TTE displaying the morphology of the occluder at 12 months. **(F)** Color doppler TTE showing left-to-right shunting at the lower border of the occluder 12 months after occlusion.

Transthoracic echocardiography was mainly performed in the apical 4-chamber view to visualize the degradation of the occluder, with the double atrial view under the xiphoid process as the supplementary view. The width and thickness of the occluder were assessed to reflect the changes in the size and overall height of the umbrella disc. The degradation of the umbrella disc was reflected by the change in the diameter of the umbrella disc, and the degradation of the thickness was reflected by the change in the overall height of the occluder. The diameter and thickness of the occluder umbrella disc together represented the volume change of the occluder (i.e., total degradation). Since all the devices were symmetrical occluders, we compared the diameters by measuring only the discs showing the complete side. The morphology of the occluder was unclear in 14 cases 6 months after occlusion, and only a hyperechoic mass was visible at the fossa ovalis. Considering the gradual degradation of the umbrella disc of the occluder, the diameter of the umbrella disc and the overall thickness of the occluder were both reduced compared to those seen 48h after occlusion. Residual occluder umbrella disc echogenicity was visible in two patients (case 3 was a patient with ASA wherein a 30/30 mm occluder was employed, and case 9 was a patient with migraine wherein a 24/24 mm occluder was employed). In case 11 (PFO combined with ASA), a residual shunt was found in the lower edge of the occluder by color Doppler TTE, and the shunt bundle was 2.0 mm wide. At 12 months postoperatively, the hyperechoic mass of the device in the middle of the atrial septum became markedly smaller in extent, including the diameter of the umbrella disc and thickness of the occluder, compared to that seen 6 months postoperatively (Figure 3). Cases 3, 4, and 7 (PFO combined with ASA) had large heterogeneity in the data regarding the diameter and thickness of the umbrella disc of the occluder owing to the implantation of a larger occluder. Therefore, the data of these three cases were excluded from the statistical analysis. However, these large occluders became markedly smaller over time as well.

**Figure 4:**
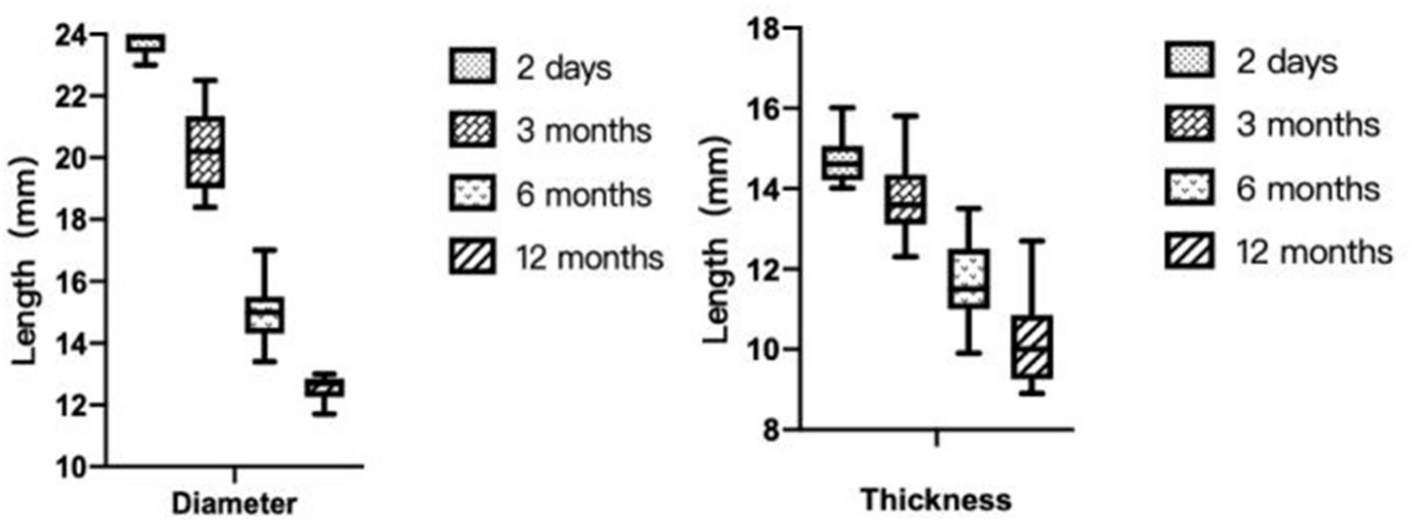
The diameter and thickness change of the occluder after closure.

In Phase I clinical trial, Zhang et al. showed that the diameters of the left and right umbrella discs of the occluder decreased significantly at 3, 6, and 12 months compared to those seen on the second day after occlusion (*p* < 0.05). At 12 months after surgery, the thickness was significantly reduced compared to those at the first three time points (*p* < 0.01 in all cases) (16). This finding is consistent with the results of our study (Figure 4).

A 2.0 mm residual shunt continued to be visible on color Doppler TTE at the lower edge of the occluder in case 11, 12 months after the occlusion (Figure 3).

##### Transesophageal echocardiography

In all patients having PFO combined with ASA, ASA regained prominence when the occluder umbrella disc was degraded 6 and 12 months postoperatively compared to that seen earlier. The size of the ASA at 12 months after occlusion was significantly reduced in comparison to that before occlusion (tumor base, 10.6 ± 1.34 vs. 28.4 ± 1.14 mm, *p* = 0.007; tumor height, 6.12 ± 2.29 vs. 15.82 ± 5.16 mm, *p* = 0.016).

**Figure 5:**
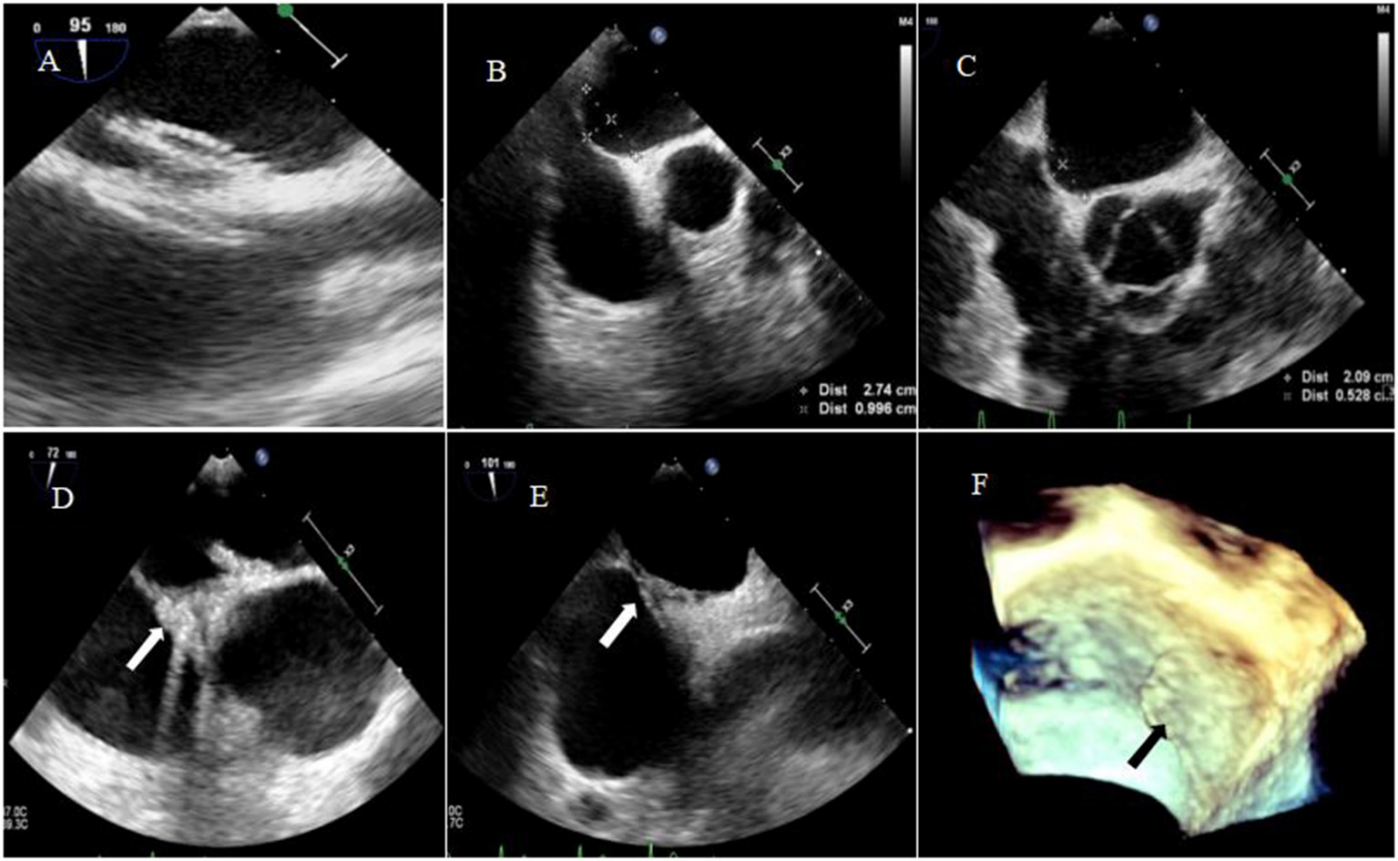
Change in ASA before and 12 months after occlusion; 2D-TEE showing the occluder during occlusion and 3D showing the occluder 12 months after occlusion. **(A)** TEE showing a grid-like structure of the occluder during occlusion; **(B)** 2D-TEE demonstrating ASA size before occlusion; **(C)** 2D-TEE showing the size of ASA reduced in the same patient; **(D)** 2D-TEE showing the long tunnel occluder; **(E)** 2D-TEE showing double umbrella disc occluder; **(F)** 3D-TEE showing complete endothelialization of the umbrella surface.

Twelve months after occlusion, TEE revealed that the interlayer membrane was completely intact between 70° and 100°. The overall direction of the interlayer membrane was along the original foramen ovale tunnel and it filled the tunnel. The left and right atrial surfaces of the interlayer membrane were attached to the left and right atrial sides of the original foramen ovale tunnel in a parallel manner. If the preoperative PFO was a long tunnel, the interlayer membrane had an inclined “femoral shape.” If the preoperative PFO tunnel was short, the interlayer membrane had a small double-disc shape. In addition, after 12 months of occlusion, TEE showed that the smaller umbrella disc was flat and smooth and the grid-like structure completely disappeared, which was completely different from the grid-like umbrella disc shape in two cases of simple TEE-guided occlusion. Therefore, we speculated that the occluder was endothelialized. While definitive endothelialization requires anatomical and histological evidence, this was difficult to achieve here. It could only be sampled and analyzed in patients requiring open-heart surgery for other subsequent problems (Figure 5).

### Residual RLS

No RLS was found in the four patients with left-to-right shunts visible at the atrial level on preoperative TTE. However, a trace residual left-to-right shunt was observed on color Doppler in case 11.

After 6 months, all patients underwent cTCD to screen for RLS. They were all grade 0 at rest. After the Valsalva maneuver, they were grade 0 in 1 case, grade I in 13 cases, grade II in 1 case (case 7), and grade III in 1 case (case 11). After 6 months, the success rate of occlusion was 87.5%.

**Figure 6:**
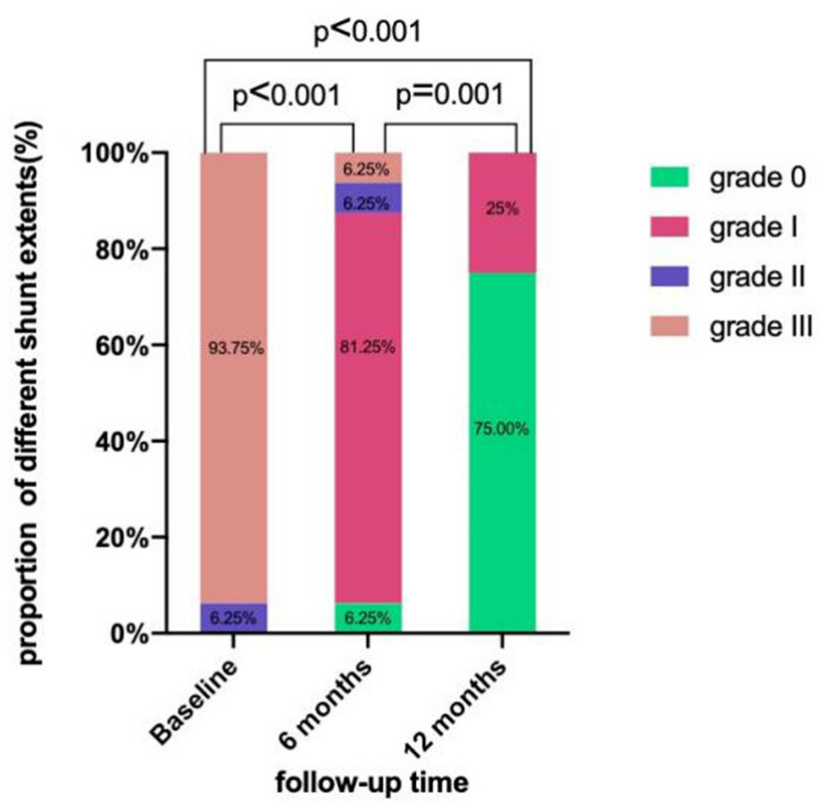
cTCD showing RLS preoperatively, 6 months, and 12 months postoperatively.

Furthermore, all patients underwent cTCD 12 months postoperatively, with 16 cases of resting-state grade 0 shunt. After the Valsalva maneuver, 12 cases had grade 0 and 4 cases had grade I shunts. Therefore, it indicates that the overall success rate of occlusion 12 months after the operation was 100% (Figure 6).

## Discussion

The main results of this study are as follows: (1) the biodegradable occluder, developed by Shanghai Mallow Medical Instrument Co., Ltd., China for the treatment of PFO *via* catheter occlusion, showed an immediate success rate of 100%, high safety, and no serious intraoperative or postoperative occlusion-related complications; (2) the occluder gradually degraded after occlusion, such that most of the umbrella disc was degraded 6 months after occlusion, as demonstrated by TTE; (3) although all patients had anatomically high-risk PFOs, the success rate of complete occlusion based on cTCD results at 12 months after occlusion was 100%, indicating that the degradable occluder performed well in general; and (4) the overall treatment efficacy in patients with migraine 12 months after PFO occlusion was 83.3%, and 80% of patients with cerebral infarction did not have a recurrent stroke.

In recent years, for patients with high-risk PFO, active treatment is generally recommended, and transcatheter interventional occlusion therapy is presently the primary treatment method.

Many studies have divided PFO into simple and complex types according to its anatomical structure and atrial septal characteristics. Complex PFOs are defined as those with a long tunnel (≥8 mm), or with a combined ASA complex lesion, or with secondary septal over thickness (> 10 mm), combined lipomatous hypertrophy of the interatrial septum, or excessive Eustachian valve or Chiari's network, or with multiple outlets on the left atrial side, or with aortic root dilatation causing anatomical abnormalities. The definition of high-risk PFO also varies among different studies. The high-risk factors for PFO anatomy in the DEFENSE-PFO study were the presence of the following: merged ASA (bulge distance ≥ 15 mm), large atrial septal activity (atrial septal protrusion ≥ 10 mm to either atrium), or large PFO (the maximum gap between primary and secondary septum ≥ 2 mm) (17). In another study, high-risk PFOs were defined as those with two or more of the following criteria: a PFO tunnel ≥ 10 mm, large atrial septal mobility (swing range ≥ 5 mm), the presence of Eustachian valve or Chiari's network, a large RLS after Valsalva maneuver, and a PFO with an angle ≤ 10° to the inferior vena cava (18). Using a combination of these diagnostic criteria, all patients in our study had anatomically high-risk PFO.

In terms of PFO diagnosis, preoperative research revealed that while cTTE examinations were influenced by many factors, its diagnostic specificity for RLS was 97–100% (19). RLS caused by PFO often presents as transient and small-volume shunts. The classification standard for cTEE to determine RLS is the same as that for cTTE. However, because TEE is usually performed under local anesthesia on an outpatient basis in China, it is difficult for patients to perform the Valsalva maneuver effectively. The detection and flow across the shunt may be underestimated on cTEE, as shown in a previous study by Gonzalez-Alujas et al. (20). However, cTEE has the ability to accurately determine the source and characteristics of RLS. As such, a preoperative cTEE examination was employed in our study. In addition, based on previous studies, we adopted the modified Valsalva maneuver while conducting a TTE, which is more advantageous in the detection of RLS (21).

In recent years, a number of randomized controlled studies on PFO occlusion for stroke prevention showed that it greatly reduced the risk of recurrent stroke (17, 22–24). The 2021 consensus of Chinese experts noted that for patients between 16 and 60 years of age, who had thromboembolic cerebral infarction with PFO and no other etiology of stroke, PFO was accompanied by ASA or a large volume of RLS, or PFO with a diameter ≥ 2 mm; transcatheter closure was a Class I and Level A indication, with a good preventive effect on recurrent stroke (25). The incidence of PFO in patients with migraine is 40–60%, which is much higher than that of the general population. An increasing number of studies have suggested that migraine with aura might be associated with PFO, particularly when there is a large volume of RLS. PFO might serve as a pathway for thrombus, platelet aggregates, serotonin, and other substances to enter the systemic circulation to induce migraine. Interventional occlusion of the PFO may be an important treatment option for migraine. There have been randomized controlled trials on the interventional treatment of PFO and migraine such as MIST, PREMIUM, and PRIMA, and although the research endpoint has not yet been reached, many observational studies have found that some patients with migraine benefited from the interventional treatment of PFO (12, 26, 27). In 2021, a JACC study found that 87% of patients had a migraine load reduction by > 50% and symptoms were reduced in both aura and non-aura groups. Migraine completely resolved in 48% patients (28), which is consistent with the findings of our study. For dizziness of unknown cause, few studies have shown a potential correlation between dizziness and PFO, and that dizziness in these patients could be relieved after transcatheter closure. However, there is currently no specific mechanism that could explain dizziness in patients with PFO (29).

The limitations of the current PFO occluder technology include the large size of the occluder, permanent implants that prevent trans-septal access to the left atrium for any subsequent treatment of left-sided heart disease, potential intraoperative complications (such as occluder displacement or dislodgement, pericardial effusion/tamponade, and residual shunt), and possible late complications (such as arrhythmias, cardiac abrasion, atrial septal dissection, coronary artery air embolism, thrombosis of the occluder, allergic reaction, iatrogenic atrial septal defects [ASD] and infective endocarditis, and sudden death).

An optimal occluder provides a temporary bridge for cardiac self-repair. Once the defect is completely endothelialized and covered by newly formed autologous tissue, the task of the occluder is completed. Several studies have shown that 1–3 months are required for the fibrogranulation tissue to completely wrap around the interlay membrane, while endothelialization of the umbrella disc, particularly the raised section in the middle, requires 3–6 months (30, 31). Therefore, the occluder need not be present 6 months beyond the procedure. The ideal occluder should be biodegradable, with non-toxic and completely absorbable degradation products. Biodegradable occluders and the 3D/4D-printed occluders are considered a next-generation novel alternative to the traditional Nitinol occluder. The evolution of occluders from non-degradable to degradable is dependent on the material of the umbrella disc and interlay membrane. The umbrella disc material is derived from non-degradable stainless steel, cobalt alloy, and nickel-titanium alloy to degradable polylactic acid (PLA), PDO, polycaprolactone (PCL), etc. The material used for the interlayer membrane evolved from non-degradable PET, polytetrafluoroethylene (PTFE), and expanded PTFE (ePTFE) to biodegradable porcine intestinal collagen and PLA (31). Research and development of degradable occluders have been conducted worldwide for over 10 years, and more than 10–20 types of occluders have been developed successively. However, all of them have failed. Apart from providing poor supporting force, the most important factor responsible for their failure is that the absorbable material is not radiopaque, so it is necessary to add metallic markers or to design the device as a metal bracket structure, which cannot be completely degraded. These non-absorbable metallic residues fall off during degradation, inducing a series of new complications such as embolism. Therefore, research and development of fully degradable occluders were once at an impasse.

The concept of “biodegradable” was first proposed with the BioSTAR PFO-occluder (30, 32), which consists of a non-degradable frame and an absorbable collagen membrane. Subsequently, partially or completely biodegradable occluders gradually emerged, such as the Carag bioresorbable septal occluder and double BioDisk occluder, 4D printing of shape-memory polymeric occluder, and Lifetech Absnow PLLA occluder (33–36). However, in recent years, fully degradable occluders have been used in preclinical research.

Pre-clinical animal experiments with the implantable biodegradable occluder in this study demonstrated the following findings. (1) In pathological analysis, the degradation cycle of the occluder was moderate. Three months after occlusion, the umbrella disc framework disintegrated from the edge. However, the structure was still intact and the surface was covered with a continuous layer of flat-shaped endothelial cells. Six months after occlusion, the architecture of occluding device was degraded and replaced by collagen fibers with visible fibroblasts inside. The framework of the occluder was completely replaced with granulation tissue 12 months after occlusion, with a variable amount of new capillaries and fibroblasts based on the fibrous connective tissue. Concurrently, the morphology of the interlayer membrane remained unchanged. (2) The occluder had good memory properties. In this regard, the degradable silk showed good shape memory and recovered its original shape when released from the conveying device. (3) The occluder had good biocompatibility. The degradable silk relied primarily on hydrolysis, allowing it to be gradually absorbed by the body, before decomposing into carbon dioxide and water with no toxic side effects and being discharged from the body. (4) The occluder had good mechanical support. In this regard, the degradation period of silk was 12 months, ensuring that the occluder had adequate mechanical support before endothelization is completed. Moreover, the occlusion device was free from metallic markers, such that there was no possibility of the metal falling off during the absorption process and causing an embolic event.

The biodegradable PFO occluders used in this study differ from conventional metal occluders as their material is slightly soft and the procedure is slightly different. In this regard, a loading sheath of a size larger than that of the conventional occluder is often selected. The operator needs to manually assist in the process of pulling the occluder into the loader to prevent alterations in the morphological structure of the occluder due to a strong pull. The occluder should not stay in the delivery system for a long time. As such, the occluder can be loaded into the sheath after the surgical path has been established. The occlusion is subsequently completed within 6 min to ensure good recovery of the structure and shape of the occluder.

**Figure 7:**
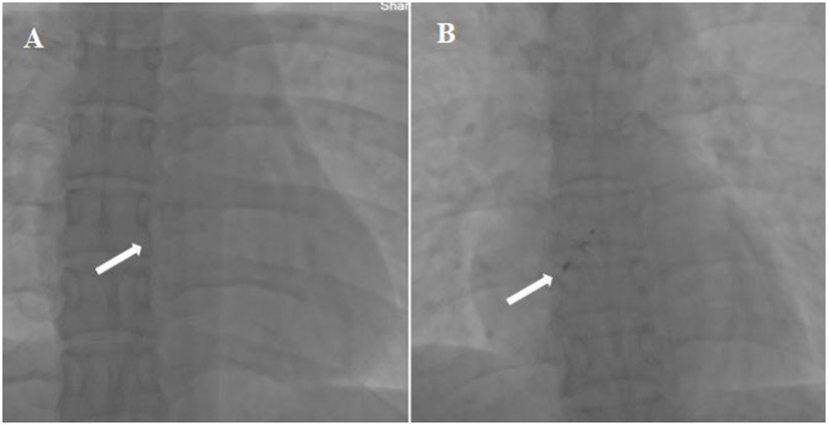
X-ray appearance of the traditional metal occluder and biodegradable occluder. **(A)** Non-visualization of degradable occluder on X-ray; **(B)** conventional metal occluder visualized under X-ray.

Since the degradable occluder is radiolucent (Figure 7), it can only be evaluated under TTE or TEE guidance during and immediately after occlusion. Studies have reported that percutaneous intervention under the guidance of TTE has routinely been performed in clinical practice for innumerable cases with a success rate of 99% and a TTE-guided rate > 95%. There were no serious complications, such as cardiac perforation, pericardial tamponade, or immediate occlusion device dropout (37). Another advantage is that X-ray exposure to medical staff and patients can be avoided.

In previous studies, the indicator of a good PFO closure with a metal occluder was its model, which was selected according to the morphological structure of the foramen ovale and anatomical characteristics of the atrial septum. The occluder completely covered the thin and wobbled section of the atrial septum and the device was attached well. Successful PFO closure was considered when the number of microbubbles in the cTCD or cTTE results 6 months after occlusion was <10. In the phase I clinical trial of the Mallow Medical biodegradable occluder, Zhang et al. showed that the success rate of occlusion was 88.6 and 95.4% at 6 and 12 months postoperatively (16), which is consistent with our results. Previous studies showed that the success rates of PFO closure with metal occluders were > 65% and > 85% at 6 and 12 months after interventional therapy, respectively (38). In another study of 219 cases with PFO that received interventional treatment with metal occluders, a success rate of 98.6% at 6 months after occlusion was observed (39). In our study, after occlusion, we uniformly selected cTCD as the screening tool for residual RLS. Assessment of the occlusion efficacy based on cTCD results was considered clinically more relevant because only the entry of microbubbles into the cranial vessels would cause migraine or stroke. Since the microbubbles or microemboli enter the cerebral circulation, the cTCD results in this study could better reflect the effect of the PFO-occluder. In case 11 of this study, a residual left-to-right shunt was seen on color Doppler TTE 12 months after occlusion. However, the cTCD was suggestive of grade I shunt, suggesting that the microbubbles were destroyed during entry into the cranial vessels.

It has been shown that PFO combined with ASA, the size of the left atrial side of the PFO, the length of the PFO tunnel, and the size of the occluder ≥ 30 mm are independent predictors of the postoperative residual shunt. RLS might be an important determinant of the improvement or recurrence of a patient's condition. Our results showed that two patients had grade II and grade III RLS 6 months after closure (cases 7 and 11, respectively). Both cases had PFO characteristics combined with ASA, and the left atrial side size of the PFO was ≥ 2 mm. In case 11, the PFO was in the shape of a long tunnel with a length of 11.5 mm. In case 7, an occluder of 34/34 mm was selected. These factors might be related to residual RLS after closure. The RLS grade was significantly reduced at 12 months compared to 6 months after occlusion, and grades II or III RLS were no longer present. This finding suggested that while the PFOs in most patients were successfully occluded 6 months after the operation, persistent endothelialization further improved the occlusion effect.

Studies have compared the random selection of atrial septal occluder (ASO) and PFO occluders in high-risk patients with PFO. One year after the operation, the residual shunt incidence rates in the ASO group and the larger PFO occluder groups were 3% and 35.5%, respectively. The lack of adequate support at the waist of a larger double-disc-shaped PFO occluder might lead to poor atrial septal attachment (40). In our study, PFO occluders of sizes 30/30, 30/30, 34/34, 24/24, and 24/24 mm were selected for five patients having PFO combined with ASA. Except for case 11, the remaining four cases showed a good plugging effect. This observation could be explained by the fact that the degradable occluder is different from the metal occluder in remaining supported on the PFO. If the anatomical structure of the PFO does not match that of the occluding device, iatrogenic enlargement of the PFO may occur, resulting in RLS. However, because most of the materials of the degradable occluding device had been degraded, it could no longer provide support. As such, the sealing effect might be better with biodegradable occluders. In our study, the 24/24 mm occluder was selected for case 11, and TTE did not detect any residual shunt in the follow-up visits at 1 month and 3 months after occlusion. TTE color Doppler findings at 6 months and 12 months after occlusion showing a residual shunt at the lower margin of the umbrella might suggest that the occluder could completely cover the PFO combined with ASA in a short period after occlusion, but, over time, anatomical features, such as long, weak, and irregular atrial septum, affect the stability of the occluder, and there is a potential for migration. Endothelization may also be relatively delayed and result in a mismatch between the material degradation time and the time required for self-regeneration and repair of the tissue at the implantation site, causing a postoperative residual shunt. For these patients, a degradable ASD occluder or a metal occluder may be preferable. Further studies with a larger sample size are required for validation.

Although some studies have suggested that residual shunts after occlusion might lead to an increased probability of recurrent stroke/TIA, it is worth noting that the clinical outcome of occlusion treatment in our study was good despite residual RLS in one patient, with an overall treatment efficiency of 85.7% in patients with migraine and no PFO-related recurrent stroke after PFO occlusion in patients with cerebral infarction. The attack duration and frequency in patients with dizziness decreased by more than 50%. In two patients (cases 10 and 12), the HIT-6 score remained high 1 year after occlusion, but cTCD revealed grade I shunt, indicating that migraine was not closely related to PFO. In addition, cases 7 and 11 still had moderate or more severe RLS 6 months after the operation. However, by careful comparison with preoperative images, we found that the number of RLS microbubbles was significantly reduced in both patients. This indicates that the mechanical obstruction caused by the occluder and its endothelialization could close most PFO structures, and potential microemboli and vasoactive materials were mostly intercepted by the occluder. According to the cTCD diagnostic criteria, RLS grade III is confirmed when a microembolus signal >25 is seen, which can be in the shape of a rain curtain or a shower. Since this quantitative grading spectrum is too wide, we believe that it was not appropriate to continue using the previous RLS grade for postoperative assessment of the blocking effect. It might be possible to perform cTTE during both pre- and post-occlusion follow-ups to assess the contrast in the echo intensity of microbubbles in the left cardiac chamber and their occupation of the left cardiac chamber. More detailed evaluation criteria must be developed for large-scale studies.

While there is a consensus on the duration of antiplatelet therapy after PFO occlusion, recent guidelines, particularly the 2019 French Society of Neurovascular and Cardiology Expert Consensus, suggested the need for enhanced postoperative antiplatelet therapy in patients with stroke with high-risk PFO, who can continue aspirin or clopidogrel monotherapy for ≥5 years after 3 months of dual antiplatelet therapy (6). However, it should be noted that long-term antiplatelet therapy can partially mask the true effect of mechanical closure of the PFO with an occluder. In this study, to minimize the additional effects of antiplatelet drugs, we focused our analysis on the clinical efficacy 6 months after occlusion (when antiplatelet drugs were stopped) and 12 months after occlusion to obtain a more accurate picture of the blocking effect.

## Limitations

This study had a small sample size and a short follow-up period. Furthermore, the sample also failed to cover all types of high-risk anatomical PFOs. TEE was not performed at every follow-up time point to assess the morphology and functionality of the degradable occluder further. TTE might have missed some information due to limitations of the acoustic window conditions. In the future, studies with a longer follow-up period and larger sample size should be conducted. It is also necessary to conduct a randomized controlled study comparing degradable occluders and traditional metallic occluders to identify the subgroup of patients who benefit from each type, thus enabling individualized and precise treatment.

## Conclusions

Our phase III single-center clinical trial of transcatheter occlusion of PFO with a degradable occluder demonstrated a high success rate and safety of the procedure. There were no serious complications, and the success rate of complete occlusion was high. The occluder performed well, even in cases of high-risk anatomical PFO. TTE could dynamically, conveniently, and accurately observe the entire degradation process of the occluder. Therefore, with an in-depth understanding of PFO pathophysiology, the biodegradable PFO occluder, developed by Shanghai Mallow Medical Instrument Co., Ltd., has wide applications. However, an individualized treatment strategy for various types of high-risk PFO and a multifactorial comparison with traditional metal occluding devices still need to be conducted in a large-scale clinical setting.

## Data availability statement

The raw data supporting the conclusions of this article will be made available by the authors, without undue reservation.

## Ethics statement

The studies involving human participants were reviewed and approved by Ethics Committee of the First Affiliated Hospital of Shandong First Medical University. The patients/participants provided their written informed consent to participate in this study.

## Author contributions

HW, XZ, and LH designed the study. HW, XZ, LH, ZL, and ML have jointly completed the operation and provided relevant materials. HW, LS, and PS collected and analyzed the data and wrote the draft. All authors read and approved the final manuscript. All authors contributed to the article and approved the submitted version.

## Funding

This study was funded by Shandong Key R&D Program (2018GSF118058).

## Conflict of interest

The authors declare that the research was conducted in the absence of any commercial or financial relationships that could be construed as a potential conflict of interest.

## Publisher's note

All claims expressed in this article are solely those of the authors and do not necessarily represent those of their affiliated organizations, or those of the publisher, the editors and the reviewers. Any product that may be evaluated in this article, or claim that may be made by its manufacturer, is not guaranteed or endorsed by the publisher.
